# Experiences of adult social work addressing self-neglect during the Covid-19 pandemic

**DOI:** 10.1177/14680173221083446

**Published:** 2022-09

**Authors:** Jill Manthorpe, John Woolham, Nicole Steils, Martin Stevens, Stephen Martineau, Jenifer Owen, Michela Tinelli

**Affiliations:** The Policy Institute, King’s College London, London, UK; Care Policy and Evaluation Centre, 4616 London School of Economics, London, UK

**Keywords:** Social work, safeguarding, adult care, risk, health and social care, ageing

## Abstract

**Summary:**

Internationally there has been much interest in the impact of the COVID-19 pandemic on the care and support of older people including those with needs arising from self-neglect and/or hoarding. During the pandemic English local authorities’ legal duties remained to respond to concerns about harm about people with care and support needs living in the community. This paper reports interviews with 44 participants working for adult safeguarding/adult protective services (APS) in 31 local authorities recruited from all English regions. Interviews took place online in November-December 2020 as the pandemic's second UK wave was emerging. Analytic induction methods were used to develop themes.

**Findings:**

Participants reported some of the variations in referrals to their services with more contact being received from community sources concerned about their neighbours’ welfare. Participants provided accounts of the local organisation of adult safeguarding services during the pandemic, including in some areas the potential for offering early help to older people at risk of harm from self-neglect or hoarding behaviour. Online inter-agency meetings were positively received but were acknowledged to potentially exclude some older people.

**Applications:**

This article reports observations from adult safeguarding practitioners about their services which may be of interest internationally and in renewing services that can sustain public interest in the welfare of their older citizens and in developing early help. The findings reflect those from children's services where online meetings are also predicted to enhance professional communications post-pandemic but similarly need to ensure effective engagement with service users and their families.

## Introduction and background

There have been many changes to social work practice and systems during the COVID-19 pandemic with child protection services reporting substantial pressures and work-related shifts in practice internationally (Baginsky & Manthorpe, 2021). The impact on adult safeguarding (the term used in England in relation to prevention of and response to harm to adults with care and support needs who are unable to protect themselves) has been less evident. Many concerns however have been expressed internationally about heightened risks of adult abuse, mistreatment, and neglect during the pandemic (see, for example, Makaroun et al., 2020; United Nations, 2020) and the impact on adult protective services (APS). Actual instances are reported to have led to ‘*a massive increase in reports of elder abuse during the pandemic’* in the United States (US) (Han & Mosqueda, 2020) and from South Africa it has been suggested that the pandemic has amplified the risks of exploitation and abuse among older people (Jacobs, 2020). An online survey of 897 US older people sheltering or shielding at home during the pandemic found the prevalence of elder abuse during the pandemic to be one in five respondents (21.3%), an 83.6% increase compared to prevalence pre-pandemic estimates (Chang & Levy, 2021). Other studies have encountered reports of increased distress from the loneliness and social isolation experienced by many older people during periods of enforced social distancing (Berg-Weger & Morley, 2020). The British Association of Social Workers (BASW) (2020) suggested that the pandemic was giving rise to several increased risk factors or concerns about adult abuse and neglect but that the ‘barriers’ created by the pandemic were also affecting the abilities of social workers to provide effective interventions.

Several explorations of social work practice with adults, beyond safeguarding are emerging, covering various jurisdictions and phases of the pandemic in their national contexts. From Tennessee US, Neely-Barnes et al. (2021) interviewed 37 directors or managers of social service or behavioural health agencies during the pandemic's ‘shut down’ period. They concluded that while the pandemic had created stress for managers and staff it had created opportunities in forcing ‘changes in the way agencies normally did business’. From England, Manthorpe et al. (2021) interviewed 22 social workers working in statutory adult services as the United Kingdom (UK)'s second wave developed in early 2021, finding that service demand had been depressed initially then increased as levels of infections and social restrictions increased, with this pattern of demand seeming to be repeated as the second wave started to take hold. Initial decreased demand for care services was ascribed to increased family support for relatives and some refusals of home care services or of moving to a care home during lockdown (the UK's first national lockdown started in March 2020). Several noted that demand was re-emerging from previous service users but there were also new referrals whose temporary care arrangements were becoming unsustainable. Some of these social workers also commented on the changes in local authority practice such as alterations to assessment and review policies that had been permitted by adopting ‘flexibilities’ of legal processes. In England, the Coronavirus Act 2020 permitted ‘easements’ or suspension of some sections of the Care Act 2014 (by June 2020, only seven LAs had registered that they were using easements; another LA had notified government that they were going to be implemented, but subsequently did not; these easement provisions were not renewed in March 2021) (see Department of Health and Social Care (DHSC), 2020; Foster, 2020). However, government Guidance accompanying the Coronavirus Act in March 2020 specifically noted that duties to promote wellbeing, and duties relating to safeguarding adults at risk, would remain in place notably under section 42 of the Care Act 2014 (Department of Health and Social Care (DHSC), 2021). Instead the Coronavirus Act guidance cautioned that it was important for safeguarding teams to be ‘*proportionate in their responses and mindful of the pressure social care providers are likely to be under’* (Department of Health and Social Care (DHSC), 2020, Guidance Annex D).

The assumptions within the government's Coronavirus Act guidance were essentially that safeguarding concerns and referrals might be expected to increase during the pandemic as a consequence of more people needing support and their support needs changing. Both may prompt concerns about safeguarding risks or incidents and affect general processes. Indeed, a group of local authority safeguarding leads (Lloyd-Smith et al., 2021) stated:

The virus and the first lockdown response challenged and completely disrupted the normal workings of Safeguarding Adult Boards (SABs) and the delivery of their statutory functions (page 134).

From England, valuable evidence about the pre-COVID-19 situation enables some possible numerical comparisons. Data from the national Safeguarding Adults Collection (SAC) compiled by NHS Digital (2020) is based on anonymised statistical returns from local authorities. Using data for the period 1 April 2019 to 31 March 2020 (just a week after the first Lockdown started in England), as a baseline helps place the COVID-19 pandemic in context by comparing numbers of safeguarding concerns raised and enquiries conducted by the local authority or its partners. The SAC is a mandatory annual return that already offered, for the purpose of our wider study (see below), a profile of the adults about whom safeguarding enquiries had been made, and the nature of the risk of abuse or neglect. Statistics of relevance to the present article were that in the pre-COVID-19 year there had been a 14.6% rise in the numbers of concerns reported to local authorities compared to the previous year.

Recognising that the SAC would not cover the COVID-19 period until its next annual reporting, the Local Government Association (LGA) (2020) made a request of local authorities to voluntarily submit their safeguarding data for the period covering January–June 2020. Comparing equivalent months in 2019 and 2020, and month by month trends, the LGA found that numbers of concerns (referrals to local safeguarding services) had dropped considerably during the initial weeks of the first UK lockdown period, but subsequently returned to and then began to exceed the previous years’ levels in June 2020. The LGA cautioned that its data reflected the local variability of safeguarding recording and the varied temporal impact of the pandemic's spread. However, it was confident about concluding that the percentage of Section 42 (Care Act 2014) enquiries related to people living at home had increased compared to those related to people living in care homes. Such reports also included a range of risks including financial scams, inability to adhere to social distance, or the operation of lockdown measures. Relevant to the present study, the LGA provided some good practice examples; one of which related to a local authority that had installed a COVID-19 ‘button’ on its main reporting system; ‘*Through this they were able to respond when data pinpointed concerns in relation to mental health, general welfare and self-neglect, as these were particularly prominent during this period’*. (Local Government Association (LGA), 2020, p. 33). While expenditure on adult safeguarding is hard to disentangle from other commitments, the Association of Directors of Adult Social Services (ADASS) (2020) estimated in June 2020 that local authorities faced an adult social care overspend of £468 m in 2020 as the pandemic had triggered deepening needs relating to new cases of safeguarding, domestic abuse, carer breakdown and hospital discharge.

This present article draws on a set of interviews with 27 Adult Safeguarding Leads and other practitioners working on safeguarding from a sample of English local authorities (the lead agency for adult safeguarding under the Care Act 2014). These were a preliminary stage in case study work on a research project (Social care responses to self-neglect and hoarding among older people: What works in practice?) whose overall aim is to improve practice understanding of what works in responses to self-neglect and hoarding among older people from a social care perspective. We are using a general, professionally-orientated definition of self-neglect which also encompasses what is sometimes called ‘hoarding behaviour’ (Department of Health and Social Care (DHSC), 2020; Wootton et al., 2019). While hoarding was recognised as a distinct disorder in the Diagnostic and Statistical Manual of Mental Health (American Psychiatric Association, 2013), the term ‘hoarding disorder’ is not widely used in adult social care or housing service in England (Magos, 2021). It is also important to note many people self-neglect but do not hoard (see Martineau et al., 2021).

The aim of this article is to explore safeguarding practice during the Coronavirus pandemic related to referrals or concerns raised about older people who were being considered at risk of harm through self-neglect and/or hoarding. As Barnett (2018) noted, this was considered an important part of social work practice with adults in ‘normal’ times, since an estimated one of five of social work cases span mental health and older people's services. A further aim is to offer a contribution to sector interest in learning *‘from the experiences of what happened in the first weeks and months of the COVID-19 pandemic to inform future practice in terms of safeguarding prevention and protection*’ (Cooper, 2020, p. 405), which may be relevant internationally.

## Methods

Using a semi-structured topic guide we undertook a set of preliminary interviews to inform later in-depth case study data collection. It was deemed feasible to gain a national picture of adult safeguarding in England by seeking a diverse sample of local authorities that could reflect different types of authority and local populations and thus be fairly generalisable. To do this, we compiled a list of local authorities in each of the nine English regions, and 99 safeguarding lead managers were identified. These managers received an email containing information about the study and an invitation to take part. This was followed if necessary 1–2 weeks later by a further email or telephone call. A minimum of three local authorities in each English region was contacted in this way, and replacement sampling used to try to ensure that the final sample contained local authorities from each region. This also ensured that all types of local authorities were represented. A small number of local authorities (5) approached declined to take part, though one of these subsequently asked to be reinvited. A further five managers expressed initial interest, but then did not respond further to attempts to establish a mutually convenient time to be interviewed. Given the level of interest in the study, the decision was made to slightly increase the sample size to 31 local authorities. Altogether 44 participants were interviewed, as several safeguarding leads asked to be interviewed with a social work colleague or a manager.

Interviews were conducted by one researcher (JW), for consistency, at a time convenient to participants and informed consent was obtained before the interview. All participants chose to be interviewed by ‘Microsoft Teams’ video-conferencing software. Whilst face-to-face interviews have long been considered the ‘gold standard’, at the time of the study COVID-19 working at home and social distancing restrictions meant they were not possible. However, conducting interviews virtually provided the opportunity to access a range of participants while also keeping participants and researchers safe (Lobe et al., 2020) although Roberts et al.’s (2021) points about researchers not seeing contexts remain relevant as workplaces can offer insights into work practices and settings. Interviews were audio-recorded with consent and data transcribed verbatim. The topic guide consisted of questions related to the local approach to organisation of safeguarding for older people who self-neglect and/or hoard, with questions added to the initial intended topics (developed pre-COVID-19) to reflect the current pandemic context and to draw out any implications for adult safeguarding overall as well as self-neglect and hoarding, in particular. The topic guide was informed by online consultation with our study Advisory Group comprising people who self-neglect and/or hoard or have family members so affected, hoarding and safeguarding service professionals, members of older people's groups and researchers interested in the topic.

Interview data were initially analysed by question areas which addressed the phenomena of interest, namely the influence of the pandemic on adult safeguarding practice with older people at risk of self-neglect and/or hoarding. While the initial coding was based on the interview questions, as a second stage new themes and codes were developed from the data, which meant that the analysis included a grounded element, in addition to prior knowledge and theory, as suggested by Meyer and Ward (2014). The research team, comprising health and social care researchers with specific interests in gerontology, was interested in factors that were shaping the experiences of practice since the start of the outbreak (deemed March 2020 in England) to the time of the interviews (November-December 2020). We aimed to explore aspects of the interview data that recounted experiences of actual practice and casework, safeguarding systems and wider adult social care contexts, and participants’ understanding of these. NVivo software was used to help manage data analysis and data saturation, in the sense that no new themes or subjects were emerging, was achieved with this sizeable number of participants. This article first discusses the ‘business of safeguarding’ including observations on referral trends during the pandemic and changes to working behaviour, then moves to community oversight, activities of ‘reaching out’, and finally inter-agency relations and working.

## Findings

Participants’ socio-demographic details are not reported in this article and their job titles are generalised as safeguarding leads or social workers with the different local authorities (referred to as LA) followed by an identifier number (eg LA1). This is to respect assurances of anonymity given to participants and their employers. Some details of cases discussed in the interviews are not given to respect confidentiality, but salient points are reported to help illustrate general discussion.

## The business of safeguarding

There was variation in the accounts of whether cases involving older people who were suspected to be self-neglecting or hoarding had increased, although many participants reported that there had been no rise in these referrals. In only a minority of local authorities had there been a continued rise in referrals (concerns reported). Others suggested that a reason for the decline in the number of cases being brought to their attention from community settings which had features of self-neglect was the difficulty of these individuals coming to the attention of public services who were no longer making regular home visits:

… those professionals that I’m talking about, for example, our (Social Housing Provider) are not doing face-to-face visits and you’re not seeing people and we’re not seeing the same reporting from professionals that we would see, our community nurses aren't going into as many people's houses, our GPs aren't, so for me it has made a difference. (LA11)

Others reported a drop in referrals overall and a marked drop in referrals covering care homes, as also found by the LGA (2020). Care homes had been placed under extreme lockdown restrictions in England, with regular inspections by the Care Quality Commission (CQC) being suspended, which may suggest less external scrutiny and many potential referrals were thought not to have been made during this period. Participants reported that they were following the proportionate approach that had been advocated in guidance, as referred to above, when thinking of contacting providers such as care homes.

## Community oversight

Other factors bought about by the lockdown and social distancing restrictions were said to be leading to more contacts with safeguarding services – some of which concerned possible self-neglect but also included more general worries about people not managing during the pandemic for a variety of reasons. Several participants suggested that this rise was the consequence of greater community oversight, such as observations in their neighbourhoods by local residents during times when most people were being asked to stay at home or in their locality, as these participants reflected:

… some of the factors that we think is impacting on those figures is that people are in their communities a lot more, people are at home and they’re noticing their neighbours a lot more, so are making those referrals and making those concerns known. (LA10)

This oversight by local citizens was in some ways replacing some of the oversight that had been built up over the years by public services. In one area, where the safeguarding lead considered there had been a substantial rise in self-neglect, examples were given of how previous general information sharing between agencies had paused with lockdown's instigated closure of local facilities or community assets:

So I think it's understandable that self-neglect has gone through the roof, and plus we’ve not had … everywhere has been closed, we’ve got a good relationship with the (national) bank, for example, they’ll ring us if they’re concerned about somebody, not from a financial perspective, but from a self-care perspective, they’ll ring us and say, blah was in today, really worried, she looked … not seen her looking like that before, so they were able to go out, but with all of those things closed, it's very much behind closed doors at the moment, I think. (LA15)

Referring to ‘hidden harms’ another participant (LA17) referred to people being brought to their attention late in the day, sometimes too late. This appeared to be a combination of people not ‘help seeking’ for fear of troubling the potentially overwhelmed authorities, obeying warnings not to contact services such as the National Health Service (NHS) and socially isolating when required to ‘shield’ officially or sometimes as a precautionary measure, and not leave the home. Such willingness to ‘stay put’ and not be a ‘trouble’ has been observed among older people living with dementia and carers (Tuijt et al., 2021) who similarly delayed help-seeking.

This may also explain the reported rise in referrals from ambulance services who were often first on the scene when a health emergency occurred.

At times, hoarding behaviour had been brought to safeguarding services’ attention when it had become obvious to citizens who were newly alert to possible neighbourhood need. Some participants reported that some people themselves were coming forward for help and support during lockdown, and services which had been helping local communities with food provision or similar had found ‘*a number of significant cases of hoarding which were absolutely not known to them' (safeguarding services)* (LA20).

## Reaching out

During lockdown many local public and voluntary services made efforts to contact those who were particularly vulnerable to infection. Participants noted that some people with hoarding disorder or similar were not included on any public sector lists of ‘vulnerable’ people who were contacted for welfare checks as they had not been considered ‘extremely clinically vulnerable’. In this context, a few participants reported ambulance services making more referrals about safeguarding concerns as they might be the only public service that was accessing someone's home and seen its condition. Overall, however, there was no consensus that hoarding had increased or worsened during the pandemic.

The local listing of and contacts with ‘vulnerable’ people who might need support especially if they were shielding meant that new connections had been made with some people who seemed to have hoarding problems and others who were potentially at risk of self-neglect. Such activity seemed to provide welcome fresh opportunity for proactive early help to address problems in ways that were sensitive and helpful.

Some early help might have been less low-key than that described above and perhaps indicated a new sense of feeling justified to make sure that people were managing in these exceptional times:

I think it's interesting that we’ve identified more as a result of COVID, and that's because, like most local authorities, what happened was we identified people with vulnerabilities and then we made direct contact with them. So if they didn't respond to a letter, we’d send them another one, and if that didn't work we’d ring, and if that didn't work we’d go and hammer on the door. So I think as a result we’ve picked up a lot of people who are hoarding and/or self-neglecting earlier, as a result of that proactive response to COVID, which has been for me one of the positives of COVID – not many – but that's definitely one of them. (LA19)

While both referrals of self-neglect and hoarding were often described as newly coming to safeguarding attention, there were reports of people receiving home care services before the pandemic but who stopped this service for fear of contagion and subsequently were unable to manage without this support. Attempts were being made to mitigate these risks by explaining, for example, that homecare workers would have personal protective equipment (PPE) such as masks.

However, other people had come to the attention of safeguarding during the pandemic as pre-existing family support had been curtailed and support had then unravelled.

## Inter-agency relations

As noted above, communications with ambulance services were reported to have increased but other agencies were sometimes softly or not so softly criticised for reducing personal contacts. Safeguarding services sometimes seemed proud of their responsiveness and face-to-face contacts:

… whilst us as a team have continued to work as normal, so we’ve continued to go into people's homes, we’ve continued to go into care homes, we’ve continued to, in effect, respond when there's safeguarding concerns, some of our partner agencies, and our biggest social housing provider in our area, they took a stance right from the very start that they weren't doing any front-facing work. (LA29)

More often, there seemed commitment to re-starting co-operation between agencies following the initial pandemic period, as illustrated by examples of sharing electronic records, for example, internally between the local authority's housing services and its social care department, or in another example between the local authority and the local fire service.

As with other reports of social work practice during COVID-19 (Baginsky & Manthorpe, 2021) development of other forms of co-operation within local authority care services and with their partners in safeguarding had accelerated during the pandemic, such as moving to online meetings, which were generally positively received.

## Discussion

These interviews reveal a picture of the variety of underlying factors behind the existing data on safeguarding adult referrals/concerns and enquiries during the pandemic. Our focus on hoarding and self-neglect in the community needs to be set in the generally greater concentration of safeguarding services on people living at home rather than in care homes during the pandemic period. Caution is needed about intra-familial abuse and self-neglect becoming the major focus of safeguarding services during the pandemic and beyond since we lack information about the situations of people living in care homes or staying in hospitals. NICE (2021) has highlighted a long-standing lack of research about the extent of and responses to self-neglect among care home residents. There is also likely to be a legacy of concerns/referrals that came to the attention of safeguarding services during the pandemic that arose from loneliness and the difficulties of social isolation that may rightly need the attention of social work services or wider community networks (see Berg-Weger & Morley, 2020) but not the specialist support of safeguarding. After our interviews, ADASS (2021) reported an increase in the complexity of people with care and support needs seeking help in the period November 2020 – March 2021 for reasons associated with domestic violence and adult safeguarding.

Participants provided a picture of being engaged in the business of safeguarding across the pandemic, not just during lockdowns or at a distance, and some contrasted this to other agencies’ behaviour. Fresh referrals or concerns were raised, with some coming from local residents who were newly alert to the ‘goings-on’ in their neighbourhoods and any people they thought needed support. While we focused on changes in the professional response, changes to the wider ‘community response’ to people in need were also deemed significant as part of the prevention of harms. As many people were involuntarily contained in their local communities during the pandemic, some were described as more likely to notice troubling behaviour on the part of others in their community and report it. Other concerns emerged from or about people known to be at risk of isolation during the pandemic or unable/unwilling to access support during this time from community assets but also family. Some family members were supporting relatives to a considerable degree (see Tuijt et al., 2021) but others had not been able to visit them and this sometimes led to sudden anxieties when visits resumed; leading to safeguarding referrals rather than to general adult social care services. This picture of varied contacts may have implications for future safeguarding practice – the greater contacts with ambulance services (already connected in many areas at managerial levels, see Stevens et al., 2017), for example, may enhance post-pandemic links. There may also be more direct contacts with safeguarding services by neighbours and other local citizens following the pandemic who are concerned about people's well-being. The implications of enhanced community surveillance may need to be considered within a more idealised picture of community cohesion and mutual aid (Tiratelli & Kaye, 2020) since there may be a thin line between ‘troubled neighbours’ and neighbours who are perceived as ‘troublesome’.

Relevant to organisational levels, participants also gave some indication of the opportunities to offer early help to people who were known to be at risk of self-neglect and/or of developing hoarding problems. While this was articulated as part of the pandemic response with national efforts to enumerate and assist the ‘shielded’ or vulnerable, these databases or population data may have the potential to be used more often to reach out to certain groups by a combination of public services, not just primary healthcare or adult safeguarding. There is increasing recognition that ‘*home might not offer the nourishing, stabilising and comforting inoculation against uncertainty that we would ordinarily expect’* (Gurney, 2020, p. 6).

Safeguarding services for adults and children alike seem to have welcomed the opportunity to have inter-professional or inter-agency meetings online and this practice seems set to continue as in other jurisdictions (as in Ireland, see Brennan et al., 2020). The implications for individuals, and how these new modes of communication reflect the principles of Making Safeguarding Personal (MSP) in which the views and contributions of the person must be articulated and respected, will need to be assessed by practitioners and managers as well as those scrutinising adult safeguarding services. As Anka et al. (2020, p. 417) noted, the MSP conversational outcome-focused approach centres on what being safe means to the person experiencing abuse and neglect and this is hard to determine online, without guarantees of privacy, or without the foundation of a trusting relationship. Similar to APS in New York City (Elman et al., 2020) our participants reported that staff were often making first contacts by telephone, but some home visits were subsequently necessary to determine risk, individual wishes, eligibility, and to set up services.

## Limitations of the study

Our study completed a limited number of interviews with safeguarding lead managers but aimed to offer a degree of representativeness by geographical region, service models, and local authority type. We also heard of joint, tri- and multi-partite organisational arrangements (some local authorities had shared safeguarding protocols and procedures for example). Thus, the sample of interview participants is likely to include a broad range of experiences and different approaches to safeguarding, allowing a view at the ‘subject from all available angles, thereby achieving a greater understanding’ (Etikan et al., 2016, p. 3). Data saturation also appeared to have been reached with these interviews as several participants made similar comments and so a variety of views and experiences were accessed. Our focus on self-neglect and hoarding means that we did not pursue information about other forms of abuse such as scams or cyber-crime related to the pandemic which have been reported as affecting the general public as well as people using care and support services or who are isolated (Ma & McKinnon, 2021).

## Conclusion

Participants’ accounts of adult safeguarding practice with a focus on hoarding and self-neglect in the context of the COVID-19 pandemic at the end of 2020 reveal mixed and changing calls on their services. There seemed to be increased contacts by ‘concerned’ individuals that may have been routed to adult safeguarding since other avenues were less easy to access or were under other pressures. The implications of this for safeguarding services are that rising numbers of concerns or referrals may have taken up much of their activities as there were less opportunities to appropriately re-route such early contacts. Like other social work agencies, usage of online communications had been accelerated and assisted inter-agency working. These may well become widespread practice with digital safeguarding assessments as suggested by Anka et al. (2020) but in cases of hoarding and self-neglect personal contacts with individuals are generally accepted as key to successful engagement (Barnett, 2018). The digital exclusion of many older people has also been highlighted during the pandemic (Seifert et al., 2021) and inter-agency safeguarding communications and policy will need to address this so not to compound multiple exclusions.
